# Supplementary material for: Archaean green-light environments drove the evolution of cyanobacteria’s light-harvesting system
Source: Nat Ecol Evol. 2025 Feb 18;9(4):599–612. doi: 10.1038/s41559-025-02637-3 (PMC11976284; doi:10.1038/s41559-025-02637-3)
Supplement: Supplementary file 1 — Supplementary Discussions 1–12, Tables 1–8 and Source data 1–8. [file 41559_2025_2637_MOESM1_ESM.pdf]

nature ecology & evolution

Supplementary information https://doi.org/10.1038/s41559-025-02637-3

Archaean green-light environments drove the evolution of cyanobacteria's light-harvesting system

In the format provided by the authors and unedited

Supplementary Information for

Archean green light environments drove the evolution of cyanobacteria's light-harvesting system

Table of Contents

A. Supplementary Discussions

1. Redox state of open oceans through Earth’s history……………………………….2
2. Concentration of iron hydroxide in cyanobacteria habitat………………………….3
3. Molar absorption coefficient of iron hydroxide……………………………………5
4. Light window for photosynthetic organisms in the Archean era…………………..6
5. Uncertainty of light window calculation…………………………………………...7
6. Impact of cyanobacterial habitat on light window analysis………………………..9
7. Formation period of green-light window…………………………………………11
8. Birth of crown-group cyanobacteria………………………………………………12
9. Evolutionary order of phycobilisome components………………………………13
10. Present-day Archean-like water environment……………………………………14
11. Correlation between light environment and phycobilin pigments………………..15
12. Diffusivities of lake and open ocean………………………………………………16

References………………………………………………………………………… 17

B. Supplementary Tables……………………………………………………………..22

C. Supplementary Figures for phylogenic analyses……………………………………29

1. Redox state of open oceans through Earth's history

The redox state of an open ocean is generally divided into four epochs[1]:

1. An anoxic environment prior to the emergence of photoferrotrophs and cyanobacteria.
2. A coexistence of oxic and anoxic environments. Oxidation of reduced iron from deeper layers through the photoferrotrophic activity and a gradually oxidizing photic zone due to cyanobacterial activity, leading to the formation of banded iron formations (BIFs).
3. An anoxic environment, rich in hydrogen sulfide, beneath an oxidized photic zone.
4. A completely oxidized environment.

Our study focuses on the second epoch, spanning from the emergence of photoferrotrophs and cyanobacteria to the Great Oxidation Event (GOE). During this era, the photic zone was progressively oxidized by cyanobacteria, resulting in a distinct chemical stratification above the anoxic layer. Reduced iron from hydrothermal vents on the seafloor oxidized at the chemocline, contributing to the formation of BIFs. Photoferrotrophs in the chemocline also facilitated the oxidation of reduced iron[2,3,4]. As the photic zone expanded, the chemocline deepened. Before the GOE, the chemocline, corresponding to the pycnocline, might have been relatively shallow.

Modern analogs of the second epoch's environment exist in various contemporary lakes and seas. In these locations, the anoxic layers below the redox boundary (chemocline) are characterized by reduced iron from hydrothermal vents, while the photic zones above the boundary are oxidized by oxygenic photosynthetic organisms. Consequently, the reduced iron is oxidized at the chemocline and precipitates,

accumulating at the sea and lake bottoms.

In the post-GOE era, the influx of oxidized sulfur compounds from the ground into the sea may have limited the supply of reduced iron to the chemocline (Canfield Ocean model[5]), reducing iron hydroxide production. The concentration of iron hydroxide in the photic zone was likely lower than in the second epoch, resulting in a different light environment.

2. Concentration of iron hydroxide in cyanobacterial habitat

Considering that the diffusivity in the open ocean during the Archean era was likely comparable to present values[6,7], we developed eight distinct models for the vertical structure of the diffusion coefficient. This approach is based on the principle that diffusivity in the deep layer beneath the pycnocline governs the influx of reduced iron to its boundary, which in turns affects the iron oxidation rate. The eight models are informed by current diffusivity measurements in the Pacific Ocean, as detailed in Supplementary Table 7. Model 1, the standard model, uses the average diffusivity in the deep layer[8], corresponding to that of the previous experiments[6,7]. Models 2 and 3 incorporate the maximum and minimum diffusivities[9,10] in this deep layer, respectively. For comparative purposes, Model 4 assumes the diffusivity in the mixed layer to be ten times lower than in Model 1. The concentration of reduced iron in the deep layer is also a crucial factor in determining its influx to the pycnocline. Based on geological and biological perspectives[3,11-13], the concentration of reduced iron is estimated to be in the range of tens to hundreds of μM. In this calculation, we selected 80 μM as the median value, following the previous studies[3]. The concentration of oxygen was set based on the median value from previous numerical simulations by the previous study[14], which is 250 nM. Models

5 and 6 apply lower oxygen concentration, which is reduced to 25 and 2.5 nM, respectively. Additionally, considering that there is uncertainty in the concentration of reduced iron, Models 7 and 8 apply ten times lower and higher than the standard value of 80 μM as an extreme case, which are observed in the Archean Ocean analogues[1].

Utilizing the parameters detailed in Extended Data Tables 6 and 7, we calculated the vertical distributions of oxygen, reduced iron, and iron hydroxide concentrations, as depicted in Extended Data Fig. 1. The concentration of iron hydroxide in the mixed layer, which serves as the habitat for cyanobacteria, is mainly determined by the influx of reduced iron to the pycnocline. The value varies between 1 and 10 μM under realistic conditions and increases up to 100 μM as an extreme case.

As anticipated, and consistent with the previous studies[2,3], the oxygenic and anoxygenic environments are clearly divided by the boundary between two distinct diffusivities, which is assumed to be coincide with the pycnocline. Importantly, the distributions of oxygen, reduced iron, and iron hydroxide are considered to be in equilibrium. This equilibrium concentration mainly depends on the diffusivity in the deep layer beneath the pycnocline, rather than within the mixed layer itself (see Extended Data Figures 1a and 1d). Because all reduced iron is consumed in the pycnocline for all the models, the concentration of iron hydroxide is determined by the influx of reduced iron to the pycnocline. Consequently, for the standard model, which applies the average value of the diffusivity in the deep layer beneath the pycnocline, the maximum concentration of iron hydroxide is approximately 10 μM.

There remains uncertainty in the concentrations of reduced iron and oxygen by a factor of 5 – 10. However, as discussed in Section 3, a tenfold change in the concentration of iron hydroxide does not significantly alter the underwater light window except for high

turbidity conditions (~ 100 μM). Moreover, even if the oxygen concentrations in oxygen oases were to rise to 17 μM, as suggested by a previous study[15], this elevation in oxygen concentration does not alter the concentration of iron hydroxide. This phenomenon occurs because all reduced iron is consumed in the pycnocline at much lower oxygen concentrations (Extended Data Fig. 1e – f). We also observed that the concentration of iron hydroxide is unaffected by variations in the pycnocline's depth or precipitation rate, both of which do not influence the influx of reduced iron. The pH range of ocean water between 6.5 and 7.5, consistent with previous studies[16,17], similarly does not impact the concentration of iron hydroxide.

Additionally, our study also explores the role of photoferrotrophs in oxidizing reduced iron. Our findings indicate that the iron hydroxide concentration remains unchanged regardless the oxidation approach of reduced iron. This observation can be explained by the consumption of all reduced iron at a reaction rate of 14 μM/day as determined by Equations (1) – (3) in the Method section of the Main text. This rate is lower than that induced by photoferrotrophs[2].

3. Molar absorption coefficient of iron hydroxide

To estimate the spectrum of incident light in cyanobacterial habitats, we measured the molar absorption coefficient of iron hydroxide and examined its dependency on both the production method and particle size. Initially, we synthesized iron hydroxides using two methods referenced in previous studies[18,19]. Extended Data Fig. 2 displays the molar absorption coefficient of iron hydroxide across different wavelengths. Notably, the molar absorption coefficient is initially high after production but diminishes over time. This variation is particularly pronounced in the ultraviolet range, while in the visible spectrum,

the molar absorption coefficients are relatively consistent.

Our findings reveal that the molar absorption coefficient in the ultraviolet spectrum is significantly influenced by the particle size of iron hydroxide but remains unaffected by the synthesis method. Given that the molar absorption coefficient in the visible range shows minimal fluctuation, we utilized the coefficient value of the particle size of ~ 100 nm for calculating the visible spectrum of the light window.

4. Light window for photosynthetic organisms in the Archean era

By combining the calculated concentration of iron hydroxide with the measured molar absorption coefficient, we determined the spectrum of the light window available to photosynthetic organisms. An optimal combination of light and nutrients, which flow into the mixed layer from below the pycnocline, contribute to the formation of a layer rich in chlorophyll *a*, typically located at the pycnocline[20,21] and nitracline[22,23]. Thus, we initially set cyanobacterial habitat depths at 50 and 20 meters. The former corresponds with the typical depth of the pycnocline, and the latter with previous study[24] suggesting 20 meters is the optimal depth to avoid harmful ultraviolet radiation in the Archean era. Extended Data Fig. 3 shows the light windows at these depths. Above 10 μM of iron hydroxide concentration, photon availability is substantially restricted due to the intense absorption by both water and iron hydroxide. Given the high molar absorption coefficient of hydroxide in harmful ultraviolet light, we also considered a habitat depth of 5 meters. The right side of Extended Data Fig. 3 depicts the photon flux density at this shallower habitat depth. Predominantly, the light window ranges between 500 and 600 nm wavelengths, except under modern ocean conditions (without the presence of iron hydroxide) and in high turbidity conditions, where the maximum iron hydroxide concentration reaches 100

μM. While the modern ocean provides a broad wavelength window ranging from blue to green light, only faint red light is available near the water surface under such high turbidity.

We also examined which pigments are most efficient at absorbing the available photons in the cyanobacterial habitat. We calculated the correlations between the light window and each pigment spectrum, as shown in Extended Data Fig. 4. Our analysis reveals that, in environments other than the modern oceans (which are devoid of iron hydroxides) and the high turbidity condition, a green-absorbing pigment (PEB) is crucial for harvesting light for photosynthesis. At higher iron hydroxide concentrations, PC becomes more significant for light collection. Essentially, the underwater light environment in the Archean era, influenced by iron hydroxide, likely served as a selective pressure for photosynthetic organisms.

In conclusion, our analysis of the underwater light spectra indicates that the Archean water environments likely formed a green-light window under a broad range of conditions. An underwater green light environment during the Archean era was highly probable, as reduced iron supplied from hydrothermal vents was continuously oxidized at the pycnocline, leading to the formation of BIFs[25-28]. Except for the modern oceans devoid of iron hydroxide, the light windows closely align with the absorption spectrum of green-absorbing pigments (PEB).

5. Uncertainty of light window analysis

There are four potential factors that might have influenced the underwater light transmission spectrum in the Archean era: 1) the concentration of iron hydroxide, 2) the types of iron hydroxide, 3) the composition of the water environment, and 4) the composition of the atmosphere. First, the concentration of iron hydroxide significantly

alters the light window, as shown in Extended Data Fig. 3. Although the reaction rate between oxygen and reduced iron was fixed for this analysis, pH and oxygen concentration significantly affect the reaction rate, as indicated by previous research[29]. As pH increases, the reaction rate also increases considerably. However, due to the high reaction rate, nearly all the reduced iron entering the pycnocline is converted into iron hydroxide when the pH exceeds 6, in line with previous findings. Additionally, the reaction rate equations (1) – (3) in the main text have been validated for low oxygen concentrations ($< 10\ \mu M$). In the standard model, based on the earlier study[14], dissolved oxygen levels in the Archean Ocean were set to 250 nM. Thus, unless the pH or oxygen concentrations differ significantly from these model values, the underwater light transmission spectrum would remain unchanged.

Second, only iron hydroxide was considered in this study because small particles, just after formation, remain buoyant and affect the underwater transmission spectrum for an extended period based on the Stokes' law. As shown in Extended Data Figure 2, the molar absorption coefficient is almost independent of particle size in the visible wavelength range. However, the molar absorption coefficient also depends on aggregation with cyanobacteria[30], particle morphology, and crystallinity[31]. Additionally, various types of ferrihydrite particles likely contributed to the formation of BIFs[31], leading to uncertainties in the absorption coefficient even in the visible range. Therefore, the impact of different conditions and types of ferrihydrite particles on the underwater transmission spectrum should be investigated in future studies.

Third, the composition of dissolved ions in the Archean ocean might have been different from that of modern open oceans. However, the absorptions of these ions, including ferrous iron Fe(II), across the visible spectrum have no significant impact on

the underwater transmission spectrum[32]. On the other hand, dissolved silica may have been abundant in the Archean Ocean[28], potentially leading to the formation of silica-Fe(II) minerals through chemical reaction between silica and reduced iron. The presence of dissolved silica could enhance the concentration of reduced iron even under oxidized conditions[33], thereby reducing the concentration of iron hydroxide.

Lastly, a haze could have formed in the atmosphere due to UV-driven photochemistry during the Archean era, especially under conditions of higher biogenic methane concentrations compared to present-day Earth[34]. Since the transmittance of organic haze increases gradually across the visible spectrum, longer-wavelength light may have been more abundant near the ocean's surface[35], compared to scenarios without the haze. However, the difference in transmittance between short and long visible wavelengths is only around 10%, suggesting that the impact of organic haze on the light window would be considerably smaller than that of iron hydroxide.

6. Impact of cyanobacterial habitat on light window analysis

This study does not explicitly define cyanobacterial habitats and species for calculating the underwater light spectrum. Although the analysis was based on the conditions similar to those in open oceans, such as diffusivity and the depth of the pycnocline (Supplementary Tables 6 and 7), the light-window analysis is not limited to open oceans but is also applicable to coastal areas and freshwater environments. A recent study[36] suggests that BIFs may have formed in freshwater environments, indicating that reduced iron could have been abundant throughout water environments in the Archean era. This scenario aligns with the fact that the emergence of crown-group cyanobacteria coincides with the formation period of continental crust[37,38] (see also Supplementary Discussion 8).

On the other hand, the concentration of iron hydroxide is expected to be lower in freshwater environments due to lower diffusivity in lakes[39]. However, as shown in Extended Data Figure 3, a green-light environment can still form under a lower concentration of iron hydroxide (~1 μM), consistent with studies on light conditions in Archean-like lakes[1]. Therefore, regardless of cyanobacterial habitats, crown-group cyanobacteria may have evolved under green-light environments.

This light-window analysis also applies to both planktonic and benthic cyanobacterial communities. Microbial mats in benthic environments grow under stable light conditions because the distributions of oxygen, reduced iron, and iron hydroxide remain in equilibrium (Supplementary Discussion 2). In contrast, the light environments for planktonic cyanobacteria fluctuate over short periods, as habitat depth changes due to oceanic diffusion, currents, and tides. However, since the light spectrum is restricted to green light a few meters below the surface in the presence of iron hydroxide, planktonic cyanobacteria are predominantly influenced by the green-light window. Based on these considerations, specific cyanobacterial habitats and species were not set for this analysis.

These assumptions were also applied to the experiments on the natural selection of phycobilin pigments. We selected *Gloeobacter violaceus* PCC7421 and *Synechococcus elongatus* PCC 7942 as model species. While these species are extant and are defined by their divergence time and habitat, it remains uncertain if they represent species that dominated during the Archean era. However, as discussed above, the choice of cyanobacteria as model species does not affect the conclusions of this study.

7. Formation period of green-light window

There are three possible scenarios to form a green-light window during the Archean era, depending on how to oxidize reduced iron supplied from thermal vents and lands: 1. indirect oxidation of reduced iron by oxygenic photosynthesis in cyanobacteria[25,27], 2. direct oxidation of reduced iron by photoferrotrophs[26-28], and 3. photochemical oxidation of reduced iron by UV radiation[40]. In the third scenario, the green-light window could have started forming around the time oceans emerged. In the first and second scenarios, the timing of the green-light environment's formation depends on the appearance of photosynthetic organisms – cyanobacteria and photoferrotrophs.

Based on the geological records, the presence of BIF, which is observed as early as 3.7 billion years ago[41,42], implies the high abundance of Fe(II) ions throughout the Archaean[3,11-13] and hence the precondition for the green-light window was already in place long before the GOE. Photoferrotrophs are hypothesized to have played an important role in the formation of the initial BIF[43], suggesting their emergence prior to the evolution of cyanobacteria. Hence, the green-light window induced by photoferrotrophs (scenario 2) may have been present already in the early Archean, regardless of the significance of scenario 3, which could have induced the green-light window even earlier. In turn, stromatolites, which are fossilized microbial colonies presumably produced by phototrophs, are similarly present in geological successions as early as 3.5 billion years ago[44,45]. This is also consistent with the geological records that gradually oxidized reduced Fe(II) ions in pre-GOE surface waters (both marine and freshwater environments)[46-49]. Although this may suggest the presence of cyanobacteria, as calculated by some molecular clock analyses, the argument is far from conclusive. Yet, several lines of geochemical evidence suggest the oxidation of surface waters by 3 billion

years ago (scenario 1)[42,44-49]. Consistently, the emergence of at least stem-group cyanobacteria before the GOE is generally agreed among recent studies[41-47->50-56].

Overall, while the green light window may have emerged as early as 3.7 billion years ago through the oxidation of reduced iron by photoferrotrophs and/or UV radiation, before the emergence of cyanobacteria, oxygenic photosynthesis further intensified this process from ~3 billion years ago. However, it is important to note that our hypothesis about phycobilisome evolution stands independently of these temporal considerations of the green-light window.

8. Birth of crown-group cyanobacteria

Intriguingly, the Meso- and Neoarchaean that were the major period for phycobilisome evolution in our hypothesis correspond to the formative age of continental crusts[37,38], which enhanced the abundance of Fe(II) ions and BIFs[3,11]. Because the influx of reduced iron Fe(II) might determine the concentration of iron hydroxide in the cyanobacterial habitat (Supplementary Discussion 2), the evolution of cyanobacteria that possessed the complete set of phycobilisomes – i.e., crown group – may have been driven by such a geological setting that intensified the formation of the green-light window. The expansion of terrestrial-related environments, including shallow water, intertidal, and freshwater regions, through continental crust formation may have provided new ecological niches for crown-group cyanobacteria that could utilize a wide range of light wavelengths, owing to once green-specialized but now versatile phycobilisomes outside the green-light window, in particular after the formation of the ozone layer, following the GOE. The apparent dominance of freshwater species in early-branching crown-group cyanobacteria[57-59] possibly echoes this historical narrative of cyanobacterial evolution.

Underwater light environments are dependent on the depth and also the concentrations of gilvin and tripton (dissolved and particulate organic matter)[60,61] and hence the light availability for cyanobacteria differ between ecological niches[61,62]. While phycobilisomes remain important light-harvesting systems for cyanobacteria in modern environments, the triplet of APC, PC and PE is not the exclusive form of existence for phycobilisomes anymore, as indicated by the loss of PE in some cyanobacteria (Fig. 3c).

9. Evolutionary order of phycobilisome components

Our phylogenetic analyses as well as previous studies[63,64] consistently suggest that the APC core membrane linker ApcE branched off first and then other APC subunits (ApcABDF), before the evolution of PC and PE, and this presumably reflects the evolutionary order of phycobiliproteins (Fig. 3a), as discussed in the main text. While APC and PC are nearly ubiquitous in cyanobacteria, PE is distributed sporadically in the phylum and thus the presence of PE in the common ancestor of crown-group cyanobacteria was not conclusive in previous studies. However, in our present study, PC- and PE-associated proteins (i.e., phycobilin synthases, rod linkers, and lyases) always form sister clades in their phylogenetic trees, suggesting a comparably ancestral origin of PE (Fig. 3b). Additionally, in many cases, early-branching cyanobacteria (e.g., *G. violaceus* PCC7421, *S.* sp. PCC 7336) occupy basal positions for PE, PC, and APC, excluding highly divergent marine planktonic species (Extended Data Fig. 9). Hence, it is likely that PE was already present in the common ancestor of crown-cyanobacteria, along with PC and APC.

In turn, the phycobilin PCB, which attach to APC and PC, requires a single enzyme *pcyA* for its biosynthesis from biliverdin IXα, while PEB, which attach to PE,

requires two enzymes *pebAB*, starting from the same substrate[65]. It is also important to note that the ultimate origin of phycobiliproteins may or may not have been associated with photosynthesis, as suggested in a previous study[66]. *PcyA* and *pebAB* are homologous to each other and the *pebAB* genes likely evolved by the duplication of an ancestral *pcyA*-like gene (Fig. 3b). Since the presence of PC and PCB is a prerequisite for the functionality of PE and PEB, the PC and PCB pair probably evolved earlier than the PE and PEB pair. The evolution of phycobilin pigments was probably a functional offshoot of chlorophyll biosynthesis that shares the intermediate protoporphyrin IX[67], in response to the rise of oxygen since biliverdin IXα is synthesized from protoporphyrin IX by oxygen-dependent heme oxygenase[68,69].

Our experiments using genetically-modified *S. elongatus* PCC 7942, which lacks PE but produces PEB, show that PEB efficiently transfers green light energy to PC (Fig. 2d). This suggests that PEB is attached to PC as also inferred in a previous study using *Synechococcus* sp. PCC 7002[64]. In fact, some cyanobacteria are known to have PC that contains PEB[69,70]. Hence, PC can accommodate both PCB and PEB. The evolution of PEB would have promoted the functional or structural specialization of PC towards PEB, resulting in the evolution of PE and ultimately modern phycobilisomes.

10. Present-day Archean-like water environment

Environments resembling the Archean era's redox conditions still exist on present-day Earth, such as Lake La Cruz[71], Lake Paul[38], Lake Matano[3], Lake Pavin[72], and the Red Sea[73]. The distinct boundary layer promotes the production of iron hydroxides in the pycnocline (chemocline), which is largely consistent with observations in these current, similar environments[1].

Although some differences exist between the Archean era and these current analogues (Supplementary Discussion 11), we discuss the contemporary light environment analogous to that of the Archean era, characterized by the widespread presence of iron hydroxide across the sea surface. Near the hydrothermal vents around Iwo Jima in the Satsunan Islands, iron hydroxide spreads into the mixed layer. Below a depth of 5.5 meters, a distinct green light environment forms, as shown in Extended Data Fig. 5c. The concentration of iron hydroxide is approximately 10 μM, which is consistent to the underwater light environment at this concentration, as indicated by the solid orange line in Extended Data Fig. 5d. This concentration of iron hydroxide around Iwo Jima, approximately 10 μM, is consistent with that of the standard model (Extended Data Fig. 1a), suggesting that the light environment mirrors that of the Archean Ocean.

A green light environment is also formed even in lakes with even minor dispersion of iron hydroxide[1]. Given this, it appears highly probable that a green light environment is a universal feature in areas where iron hydroxide forms.

11. Correlation between light environment and phycobilin pigments

We measured the correlation between light environments and photosynthetic pigments in natural habitats. As discussed in Supplementary Discussion 10, the green-light environment is formed at the depth of 5.5 meters. By the fluorescence excitation spectrum of PE and flow cytometry, we observed phytoplankton harboring phycoerythrin pigments (PEB and PUB) in this region (Extended Data Fig. 5). Remarkably, the per-cell abundance of PE within cyanobacterial communities is notably higher in the distinctive green light environment at a depth of 5.5 meters compared to the surface layer. In conclusion, we observed excessive photosynthetic organisms harboring phycoerythrin pigments (PEB

and PUB) under green-light environment (Extended Data Fig. 5), compared to of the surface water.

12. Diffusivities of Lake and Open Ocean

The concentration of iron hydroxide formed in the chemocline varies with the degree of diffusion. In stratified layers with low diffusion, colloidal and particulate iron hydroxides remain in the chemocline and are removed by precipitation. The concentration of iron hydroxide is thus determined by a balance between its formation rate and rate of precipitation. In environments with low diffusion coefficients (10^{-7} – 10^{-8} m^2/s) and stratification, iron hydroxides are confined within the chemocline[74].

In highly diffusive photic zones of open oceans, the concentration of iron hydroxide would be uniformly constant, and iron hydroxide is predominantly removed from the photic zone through diffusion. This suggests that in environments with high diffusion, the distribution of iron hydroxide concentration is governed not by precipitation but by a balance between its formation rate and oceanic diffusivity. In the mixed layer above a pycnocline, the diffusion coefficients are generally between 10^{-3} and 10^{-4} m^2/s [9,74], indicating a short timescale for uniform substance mixing. Conversely, in deeper layers below the pycnocline, the diffusion coefficient is typically 10^{-5} – 10^{-6} m^2/s [8,9]. Even the smallest diffusion coefficients in open oceans are 100 to 1000 times larger than those of lakes.

References

1. Camacho, A., Walter, X. A., Picazo, A. & Zopfi, J., Photoferrotrophy: remains of an Ancient Photosynthesis in modern environments. *Front. Microbiol.* **8**, 323 (2017).
2. Kappler, A., Pasquero, C., Konhauser, K. O. & Newman, D. K., Deposition of banded iron formations by anoxygenic phototrophic Fe(II)-oxidizing bacteria. *Geology* **33**, 865 – 868 (2005).
3. Crowe, S. A. et al. Photoferrotrophs thrive in an Archean ocean analogue. *Proc. Natl. Acad. Sci. USA.* **105**, 15938 – 15943 (2008).
4. Ozaki, K., Thompson, K. J., Simister, R. L., Crowe, S. A. & Reinhard, C. T. Anoxygenic photosynthesis and the delayed oxygenation of Earth's atmosphere. *Nat. Commun.* **10**, 3026 (2019).
5. Canfield, D. E. A new model for Proterozoic ocean chemistry, *Nature* **396**, 450 – 453 (1998).
6. Oeschger, H., Siegenthaler, U., Schotterer, U. & Gugelmann, A. A box diffusion model to study the carbon dioxide exchange in nature, *Tellus* **27**, 97 – 192 (1975).
7. Francois, L. M. & Gerard, J.-C. Reducing power of ferrous iron in the Archean Ocean, 1. Contribution of photosynthetic oxygen, *Paleoceanogr. Paleoclimat.* **1**, 355 – 368 (1986).
8. Ledwell, J. R., Watson, A. J. & Law, C. S. Evidence for slow mixing across the pycnocline from an open-ocean tracer-release experiment. *Nature* **364**, 701 – 703 (1993).
9. Itoh, S., Kaneko, H., Kouketsu, S. et al. Vertical eddy diffusivity in the subsurface pycnocline across the Pacific. *J. of Oceanogr.* **77**, 185 – 197 (2021).
10. Qiu, B., Hacker, P., Chen, S. et al. Observations of the subtropical mode water evolution from the Kuroshio extension system study, *J. of Phys. Oceanology* **36** 457 – 473 (2006).
11. Konhauser, K. O., Amskold, L., Lalonde, S. V. et al. Decoupling photochemical Fe(II) oxidation from shallow-water BIF deposition, *Earth and Planetary Science Letter* **258**, 87 – 100 (2007).
12. Swanner, E. D., Mloszewska, A. M., Cirpka, O. A. et al., Modulation of oxygen production in Archean oceans by episodes of Fe(II) toxicity. *Nat. Geosci.* **8**, 126 – 130 (2015).
13. Konhauser, K. O. et al. Iron formation: A global record of Neoarchean to Palaeoproterozoic environmental history. *Earth-Science Reviews* **172**, 140-177 (2017).
14. Olson, S. L., Kump, L. R., Kasting, J. F. Quantifying the areal extent and dissolved oxygen concentrations of Archean oxygen oases. *Chemical Geology* **362**, 35 – 43 (2013).
15. Catling, D. C. & Zahnle, K. J. The Archean atmosphere. *Sci. Adv.* **6**, aax1420 (2020).
16. Krissansen-Totton, J., Arney, G. N. & Catling, D. C. Constraining the climate and ocean pH of the early Earth with a geological carbon cycle model, *PNAS* **115**, 4105 – 4110 (2018).
17. Halvey, L. & Bachan, A. The geologic history of seawater pH, *Science* **355**, 1069 – 1071 (2017).
18. Babin, M. & Stramski, D. Variations in the mass-specific absorption coefficient of mineral particles suspended in water. *Limnol. Oceanogr.* **49**, 756 – 767 (2004).

19. Nichols, J. B., Kreamer, E. O. & Bailey, E. D. Particle size and constitution of colloidal ferric oxide. II. Dialysis and aging. *J. Phys. Chem.* **36**, 505-514 (1932).
20. Herbland, A. & Volturiez, B. Hydrological structure analysis for estimating the primary production in the tropical Atlantic Ocean, *J. Mar. Res.* **37**, 87 – 101 (1979).
21. Cullen, J. J. Subsurface chlorophyll maximum layers: enduring enigma or mystery solved?, *Ann. Rev. Mar. Sci.* **7**, 207 – 239 (2015).
22. Brown, Z. W., Lowry, K. E., Palmer, M. A. et al. Characterizing the subsurface chlorophyll a maximum in the Chukchi Sea and Canada, *Basin. Deep. Res. Part II Top. Stud. Oceanogr.* **118**, 88 – 104 (2015).
23. Martin, J., Tremblay, J.-E., Gagnon, J. et al. Prevalence, structure and properties of subsurface chlorophyll maxima in Canadian Arctic waters, *Mar. Ecol. Prog. Ser.* **412**, 69 – 84 (2010).
24. Cockell, C. S. Ultraviolet radiation and the photobiology of Earth's early oceans. *Orig. Life Evol. Biosph.* **30**, 467 – 500 (2000).
25. Cloud, P. E. Jr. Atmospheric and hydrospheric evolution on the primitive Earth. *Science* **160**, 729 – 736 (1968).
26. Widdel, F. et al. Ferrous iron oxidation by anoxygenic phototrophic bacteria. *Nature* **362**, 834 – 836 (1993).
27. Beukes, N. Early options in photosynthesis. *Nature* **431**, 522 – 523 (2004).
28. Konhauser, K. O. et al. Iron formation: A global record of Neoarchean to Palaeoproterozoic environmental history. *Earth-Science Reviews* **172**, 140-177 (2017).
29. Kanzaki, Y. & Murakami, T., Rate law of Fe(II) oxidation under low O2 conditions, *Geochimica et Cosmochimica Acta* **123**, 338 – 350 (2013).
30. Li, Y. Sutherland, B.R. Gingras, M.K. Owttrim, G.W. & Konhauser, K. O. A novel approach to investigate the deposition of (bio)chemical sediments: The sedimentation velocity of cyanobacteria-ferrihydrite aggregates. *J. Sedimentary Research* **91**, 390 – 398 (2021).
31. Sherman, D. M. & Waite, T. D. Electronic spectra of Fe3+ oxides and oxide hydroxides in the near IR to near UV. *American Mineralogist* **70**, 1262 – 1269 (1985).
32. Ranjan, S. et al. UV transmission in natural waters on prebiotic Earth. *Astrobiology*, **22**, 242 – 262 (2022).
33. Tostevin, R. & Sevgen, S. The role of Fe(II)-silicate gel in the generation of Archean and Paleoproterozoic chert. *Geology* **52**, 706 – 711 (2024)
34. Catlin, D. C. & Zahnle, K. J., The Archean atmosphere, *Sci. Adv.* **6**, eaax1420 (2020).
35. He, C., Radke, M., Moran, S. E., et al., Optical properties of organic haze analogues in water-rich exoplanet atmospheres observable with JWST, *Nat. Astron.* **8**, 182 – 192 (2024).

36. Heubeck, C. Beukes, N. de Kock, M. et al. BASE (Barberton Archean Surface Environments) – drilling Paleoarchean coastal strata of the Barberton Greenstone Belt. *Scientific Drilling* **33**, 129 – 172 (2024).
37. O'Nions, R. K., Evensen, N. M. & Hamilton, P. J. Geochemical modeling of mantle differentiation and crust growth. *J. Geophys. Res.* **84**, 6091 – 6101 (1979).
38. Wu, F. Zhao, G. Wilde, S. A. & Sun, D. Nd isotopic constraints on crustal formation in the North China Craton. *J. Asian Earth Sci.* **24**, 523 – 545 (2005).
39. Taillefert, M. & Gaillard, J.-F. Reactive transport modeling of trace elements in the water column of a stratified lake: iron cycling and metal scavenging, *J. of Hydrology* **256**, 16 – 34 (2002).
40. Konhauser, K.O. Amskold, L. Lalonde, S.V. Posth, N.R. Kappler, A. & Anbar, A. Decoupling photochemical Fe(II) oxidation from shallow-water BIF deposition. *Earth and Planetary Science Letters* **258**, 87 – 100 (2007).
41. Eriksson, P. G., Altermann, W., Nelson, D. R., Mueller, W. U. & Catuneanu, O. Chapter 5 - Evolution of the hydrosphere and atmosphere. *Developments in Precambrian Geology* **12**, 359 – 511 (2004).
42. Czaja, A. D. et al. Biological Fe oxidation controlled deposition of banded iron formation in the ca. 3770 Ma Isua Supracrustal Belt (West Greenland). *Earth Planet. Sci. Lett.* **363**, 192 – 203 (2013).
43. Konhauser, K.O. Hamade, T. Morris, et al. Could bacteria have formed the Precambrian banded iron formations? *Geology*, **30**, 1079 – 1082 (2002).
44. Walter, M. R., Buick, R. & Dunlop, J. S. R. Stromatolites 3,400 – 3,500 Myr old from the North pole area, western Australia. *Nature* 284, 443 – 445 (1980).
45. Allwood, A. C., Walter, M. R., Kamber, B. S., Marshall, C. P. & Burch I. W. Stromatolite reef from the early Achean era of Australia. *Nature* **441**, 714 – 718 (2006).
46. Planavsky, N. J. et al. Evidence for oxygenic photosynthesis half a billion years before the Great Oxidation Event. *Nature Geosci.* **7**, 283 – 286 (2014).
47. Eickmann, B. et al. Isotopic evidence for oxygenated Mesoarchean shallow oceans. *Nat. Geosci.* **11**, 133 – 138 (2018).
48. Robbins, L. J. et al. Manganese oxides, Earth surface oxygenation, and the rise of oxygenic photosynthesis. *Earth-Sci. Rev.* **239**, 104368 (2023).
49. Ossa, F. O. et al. Unusual manganese enrichment in the Mesoarchean Mozaan Group, Pongola Supergroup, South Africa. *Precambrian Res.* **281**, 414 – 433 (2016).
50. Fournier, G. P. et al. The Archean origin of oxygenic photosynthesis and extant cyanobacterial lineages. *Proc. R. Soc. B* **288**, 20210675 (2021).

51. Magnabosco, C., Moore, K. R., Wolfe, J. M. & Fournier, G. P. Dating phototrophic microbial lineages with reticulate gene histories. *Geobiology* **16**, 179 – 189 (2018).
52. Garcia-Pichel, F. et al. Timing the evolutionary advent of cyanobacteria and the later great oxidation event using gene phylogenies of a sunscreen. *mBio* **10**, e00561-19 (2019).
53. Schirrmeister, B. E. Gugger, M. & Donoghue, P. C. J. Cyanobacteria and the Great Oxidation Event: evidence from genes and fossils. *Palaeontology* **58**, 769 – 785 (2015).
54. Betts, H. C. et al. Integrated genomic and fossil evidence illuminates life's early evolution and eukaryote origin. *Nat. Ecol. Evol.* **2**, 1556 – 1562 (2018).
55. Shin, P. M., Hemp, J. Ward, L. M., Matzke, N. J. & Fischer, W. W. Crown group Oxyphotobacteria postdate the rise of oxygen. *Geobiology* **15**, 19 – 29 (2017).
56. Oliver, T. Sanchez-Baracaldo, P. Larkum, A. W., Rutherfold, A. W. & Cardona, T. Time-resolved comparative molecular evolution of oxygenic photosynthesis. *Biochim. Biophys. Acta* **1862**, 148400 (2021).
57. Blank, C. E. & Sanchez-Baracardo, P. Timing of morphological and ecological innovations in the cyanobacteria – a key to understanding the rise in atmospheric oxygen. *Geobiology* **8**, 1 – 23 (2010).
58. Dagan, T. et al. Genomes of Stigonematalean cyanobacteria (Subsection V) and the evolution of oxygenic photosynthesis from prokarytoes to plastids. *Genome Biol. Evol.* **5**, 31 – 44 (2012).
59. Hammerschmidt, K., Landan, G., Tria, F. D. K., Alcorta, J. & Dagan, T. The order of trait emergence in the evolution of cyanobacteria multicellularity. *Genome Biol. Evol.* **13**, evaa249, 2020.
60. Kirk, J. T. O. Light and Photosynthesis in Aquatic Ecosystems. *Cambridge University Press* (1994).
61. Stomp, M., Huisman, J., Voros, L. et. al. Colourful coexistence of red and green picocyanobacterial in lakes and seas. *Ecol. Lett.* **10**, 290 – 298, (2007).
62. Stomp, M. Huisman, J. Jongh, F. D. et al. Adaptive divergence in pigment composition promotes phytoplankton biodiversity. *Nature* **432**, 104 – 107 (2004).
63. Zhao, F. & Qin, S. Evolutionary analysis of phycobiliproteins: implications for their structural and functional relationships. *J. Mol. Evol.* **63**, 330 – 340 (2006).
64. Apt, K. E., Collier, J. L. & Grossman, A. R. Evolution of the phycobiliproteins. *J. Mol. Biol.* **248**, 79 – 96 (1995).
65. Dagnino-Leone, J. et al. Phycobiliproteins: structural aspects, functional characteristics, and biotechnological perspectives *Comput. Struct. Biotechnol. J.* **20**, 1506–1527 (2022).
66. Rockwell, N. C. et al. Elucidating the origins of phycocyanobilin biosynthesis and phycobiliproteins. *Proc. Natl. Acad. Sci. USA.* **120**, e2300770120 (2023).

67. Fujita, Y., Tsujimoto, R. & Aoki, R. Evolutionary aspects and regulation of tetrapyrrole biosynthesis in cyanobacteria under aerobic and anaerobic environments. *Life* **5**, 1172 – 1203 (2015).
68. Aoki, R., Goto, T. & Fujita, Y. A Heme oxygenase isoform is essential for aerobic growth in the cyanobacterium *Synechocystis* sp. PCC 6803: modes of differential operation of two isoforms/enzymes to adapt low oxygen environments in cyanobacteria. *Plant Cell Physiol.* **52**, 1744 – 1756 (2011).
69. Alvey, R. M. Biswas, A., Schluchter W. M. & Bryant D. A. Effects of modified Phycobilin biosynthesis in the cyanobacterium *Synechococcus* sp. strain PCC7002 *J. Bacteriol.* **193**, 1663 – 1671 (2011).
70. Ong, L. J. & Glazer, A. N. R-phycocyanin II, a new phycocyanin occurring in marine *Synechococcus* species. Identification of the terminal energy acceptor bilin in phycocyanins. *J. Biol. Chem.* **262**, 6323 – 6327 (1987).
71. Walter, X. A., Picazo, A., Miracle, M. R. et al. Phototrophic Fe(II)-oxidation in the chemocline of a ferruginous meromictic lake, *Frontiers in Microbiology* **5**, 713 (2014).
72. Bura-Nakic, E., Viollier, E., Jezequel, D., Thiam, A. & Ciglenecki, I. Reduced sulfer and iron species in anoxic water column of meromictic crater Lake Pavin, *Chemical Geology* **266**, 311 – 317 (2009).
73. Schmidt, M., Botz, R., Faber, E. et al. High-resolution methane profiles across anoxic brine-seawater boundaries in the Atlantis-II, Discovery, and Kebrit Deeps (Red Sea), *Chemical geology* **200**, 359 – 375 (2003).
74. Cronin, M. F., Pelland, N. A., Emerson, S. R. & Crawford, W. R. Estimating Diffusivity from the mixed layer heat and salt balances in the North Pacific, *JGR Oceans* **120**, 7346 – 7362 (2015).
75. Hopkins, J. E., Palmer, M. R., Poulton, A. J., Hickman, A. E. & Sharples, J. Control of a phytoplankton bloom by wind-driven vertical mixing and light availability, *Limnol. Oceanogr.* **9999** 1 – 24 (2021).

Supplementary Table 1. Total of correlated photon number rate with each photosynthetic pigment in visible (400 – 700 nm).

		Chl *a* [/m^2/s]	PE [/m^2/s]	PC [/m^2/s]	APC [/m^2/s]
20 m	0μM	86.6	172.83	46.63	31.26
	1 μM	10.72	88.62	29.73	18.73
	10 μM	0.56	3.33	3.47	2.49
50 m	0 μM	54.5	66.37	11.4	8.66
	1 μM	0.41	8.76	2.12	1.28
	10 μM	0	0.003	0.003	0.002
5 m	0 μM	150.24	340.03	188.52	150.33
	1 μM	87.42	275.04	171.01	137.45
	10 μM	30.96	92.14	103.35	90.86
	100 μM	8.69	3.60	16.48	20.10

Supplementary Table 2. Parameters for the Förster distance calculations.

Donor	PCB (in APC)	β-Carotene
Acceptor	Chl *a*	Chl *a*
φ_D	6.00×10^{-1}	6.00×10^{-5}
J (M^{-1} cm^3)	1.60×10^{-12}	1.35×10^{-13}
R_0^P/R_0^C	7.01	

φ_D : The fluorescence quantum yield of the donor molecule

J : The overlap between the fluorescence spectrum of the donor molecule and the absorption spectrum of the acceptor molecule

R_0^P/R_0^C : The ratio of the Förster distance of phycobilin–Chl *a* to β-carotene–Chl *a*

Supplementary Table 3. The absolute value of electronic coupling (|V|), the intermolecular distance (R), and the orientation factor (κ) of pairs of carotenoids and Chl *a* showing the largest electronic coupling in cyanobacterial PSI and PSII.

	|V| (cm^{-1})		R (Å)		κ	
	PSII	PSI	PSII	PSI	PSII	PSI
β-Carotene	319.9	494.1	10.58	6.99	0.689	0.259
Cryptoxanthin	248.1		8.32		0.268	
Zeaxanthin		269.7		16.19		2.248
3'-Hydroxyechinenone		478.9		8.00		0.420
Canthaxanthin		432.9		8.04		0.390
Echinenone		501.9		8.22		0.506

Supplementary Table 4. Electronic couplings (|*V*|) in PS I of cyanobacteria (PDB code: 5oy0).

Chromophore pair	|*V*| (cm^{-1})
Chl *a* (B-1230) - Zeaxanthin (J-4015)	255.64
Chl *a* (F-1302) - Zeaxanthin (F-4016)	269.72
Chl *a* (A-1131) - 3'-Hydroxyechinenone (I-4020)	478.86
Chl *a* (B-1211) - Echinenone (B-4006)	356.37
Chl *a* (A-1105) - *β*-carotene (A-4019)	445.33
Chl *a* (A-1105) -*β*-carotene (A-4012)	339.06
Chl *a* (A-1107) -*β*-carotene (A-4012)	494.14
Chl *a* (A-1120) - *β*-carotene (A-4001)	277.83
Chl *a* (A-1122) - *β*-carotene (A-4007)	392.64
Chl *a* (A-1124) - *β*-carotene (A-4008)	312.58
Chl *a* (A-1101) - *β*-carotene (J-4013)	282.09
Chl *a* (B-1237) - *β*-carotene (L-4019)	251.99
Chl *a* (B-1218) - β-carotene (B-4004)	326.82
Chl *a* (B-1229) - *β*-carotene (B-4014)	279.96
Chl *a* (B-1238) - *β*-carotene (L-4019)	315.73
Chl *a* (F-1301) - *β*-carotene (B-4014)	299.40
Chl *a* (L-1501) - *β*-carotene (L-4022)	328.88
Chl *a* (a-1131) - Echinenone (i-4020)	501.91
Chl *a* (b-1211) - Echinenone (b-4006)	305.71
Chl *a* (a-1105) - *β*-carotene (a-4019)	320.0
Chl *a* (a-1105) - *β*-carotene (a-4012)	348.77
Chl *a* (a-1107) - *β*-carotene (a-4012)	465.85
Chl *a* (a-1118) - *β*-carotene (k-4001)	268.64
Chl *a* (a-1122) - *β*-carotene (a-4007)	390.20
Chl *a* (a-1124) - *β*-carotene (a-4008)	259.66
Chl *a* (a-1138) - *β*-carotene (b-4014)	257.87
Chl *a* (a-1101) - *β*-carotene (j-4013)	253.59
Chl *a* (b-1218) - *β*-carotene (b-4004)	300.84
Chl *a* (b-1228) - *β*-carotene (f-4016)	254.49
Chl *a* (b-1229) - *β*-carotene (b-4014)	286.08

Chl *a* (b-1238) - *β*-carotene (l-4019)	329.01
Chl *a* (l-1501) - *β*-carotene (l-4022)	356.94
Chl *a* (1-1131) - Canthaxanthin (h-4020)	432.88
Chl *a* (2-1211) - Echinenone (2-4006)	259.94
Chl *a* (1-1105) - *β*-carotene (1-4019)	406.57
Chl *a* (1-1105) - *β*-carotene (1-4012)	335.76
Chl *a* (1-1107) - *β*-carotene (1-4012)	451.74
Chl *a* (1-1118) - *β*-carotene (8-4001)	279.06
Chl *a* (1-1120) - *β*-carotene (1-4001)	260.90
Chl *a* (1-1122) - *β*-carotene (1-4007)	404.28
Chl *a* (1-1124) - *β*-carotene (1-4008)	291.96
Chl *a* (1-1138) - *β*-carotene (2-4014)	251.09
Chl *a* (1-1101) - *β*-carotene (7-4013)	256.68
Chl *a* (2-1228) - *β*-carotene (6-4016)	273.62
Chl *a* (2-1229) - *β*-carotene (2-4014)	257.14
Chl *a* (2-1238) - *β*-carotene (0-4019)	334.45
Chl *a* (6-1301) - *β*-carotene (2-4014)	337.13
Chl *a* (0-1501) - *β*-carotene (0-4022)	373.75

The chromophore pairs with electronic couplings greater than 250 cm^{-1} in PSI of cyanobacteria are summarized. The electronic couplings were calculated using the TrESP method. The ID number of each chromophore is given in parentheses.

Supplementary Table 5. Electronic couplings (|*V*|) in PS II of cyanobacteria (PDB code: 7n8o).

Chromophore pair	|*V*| (cm^{-1})
Chl *a* (A-408) - *β*-carotene (A-409)	278.85
Chl *a* (B-614) - *β*-carotene (B-617)	319.89
Chl *a* (C-502) - *β*-carotene (C-515)	295.55
Chl *a* (C-508) - *β*-carotene (C-515)	250.41
Chl *a* (C-512) - *β*-carotene (K-103)	308.32
Chl *a* (C-514) - *β*-carotene (Z-101)	285.61
Chl *a* (a-408) - *β*-carotene (a-409)	278.80
Chl *a* (b-614) - *β*-carotene (b-617)	319.76
Chl *a* (c-502) - *β*-carotene (c-515)	295.66
Chl *a* (c-508) - *β*-carotene (c-515)	250.43
Chl *a* (c-512) - *β*-carotene (k-103)	308.31
Chl *a* (c-514) - *β*-carotene (z-101)	285.68

The chromophore pairs with electronic couplings greater than 250 cm^{-1} in PSII of cyanobacteria are summarized. The electronic couplings were calculated using the TrESP method. The ID number of each chromophore is given in parentheses.

Supplementary Table 6. Fiducial parameters used for our numerical simulations except for diffusivity.

Item	Value	References
Concentration of reduced iron in a layer below the pycnocline	80 µM	9, 25
Concentration of oxygen in a layer above the pycnocline	250 nM	14
Depth of pycnocline	50 meters	8, 75
Rate of precipitation	40 µm/min	39
pH	6.5 – 7.5	16, 17
$log k_{ox}$	13.41	29
Iron oxidation rate by photoferrotrophs	14 µM/day	2

Supplementary Table 7. Models of eddy diffusivity, reduced iron and oxygen concentrations for open ocean.

	Upper layer [m²/s]	Lower layer [m²/s]	Ratio of reduced iron concentration to its standard value, 80 µM	Ratio of oxygen concentration to its standard value, 250 nM	Reference for diffusivity
Model 1	10^{-3}	10^{-5}	1	1	9 (Upper), 8 (Lower)
Model 2	10^{-3}	10^{-4}	1	1	9 (Upper), 10 (Lower)
Model 3	10^{-3}	10^{-6}	1	1	9 (Upper), 9 (Lower)
Model 4	10^{-4}	10^{-5}	1	1	74 (Upper), 8 (Lower)
Model 5	10^{-3}	10^{-5}	1	0.1	9 (Upper), 8 (Lower)
Model 6	10^{-3}	10^{-5}	1	0.01	9 (Upper), 8 (Lower)
Model 7	10^{-3}	10^{-5}	0.1	1	9 (Upper), 8 (Lower)
Model 8	10^{-3}	10^{-5}	10	1	9 (Upper), 8 (Lower)

Supplementary Table 8. Environmental parameters in the sea area around Iwo Jima in the Satsunan Islands.

	Temperature, ºC	Salinity, ‰	pH	DO, μM	Chl *a*, μg L^{-1}	total iron, μM
0 m	24.8	34.1	6.1	160	0.11	42.3
5.5 m	24.3	34.1	7.8	220	0.62	3.0

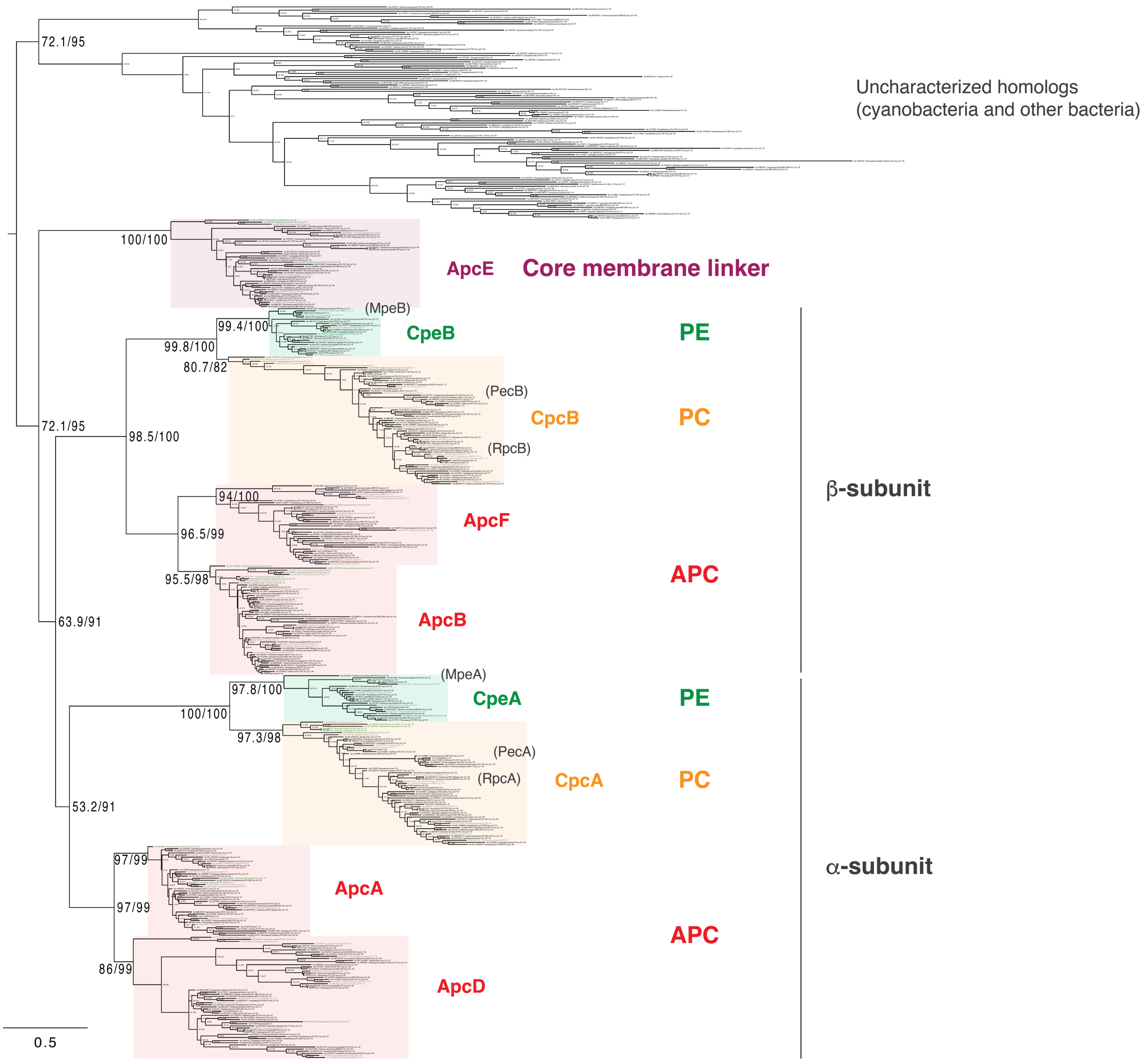

Supplementary Figure 1. Source data for Figure 1a and Extended Data Figure 9: Maximum likelihood tree of phycobiliproteins – PE (CpeAB), PC (CpcAB), APC (ApcABDF), and core membrane linker (L_{CM}) (ApcE) homologs. Green color in the annotation indicates early-branching cyanobacterial species, while grey color indicates non-cyanobacterial species within cyanobacterial clades. The support values (left for SH-aLRT; right for BS) are shown at each node.

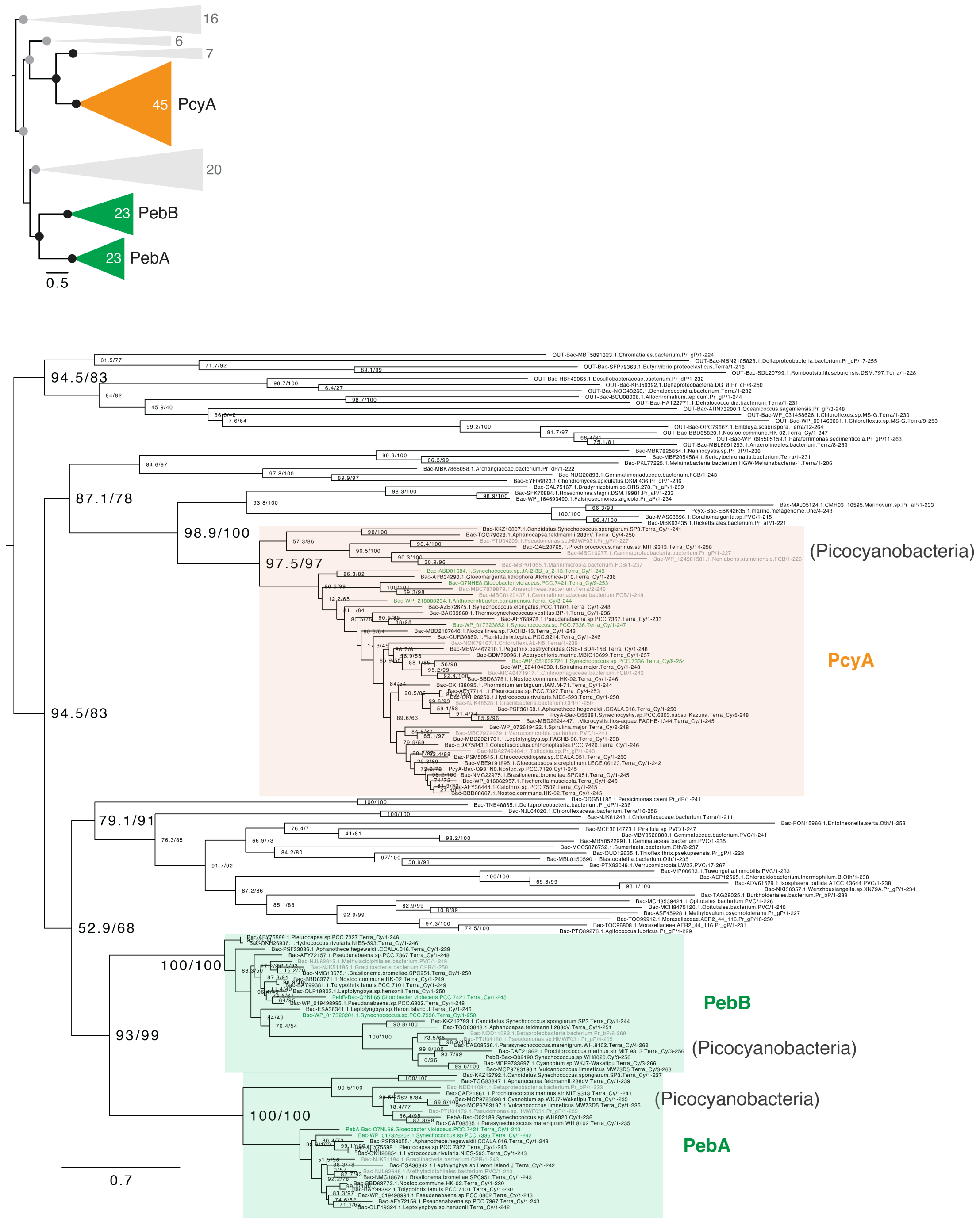

Supplementary Figure 2. Source data for Figure 3b and Extended Data Figure 9: Maximum likelihood tree of PebA, PebB, and PcyA homologs (phycobilin biosynthesis).

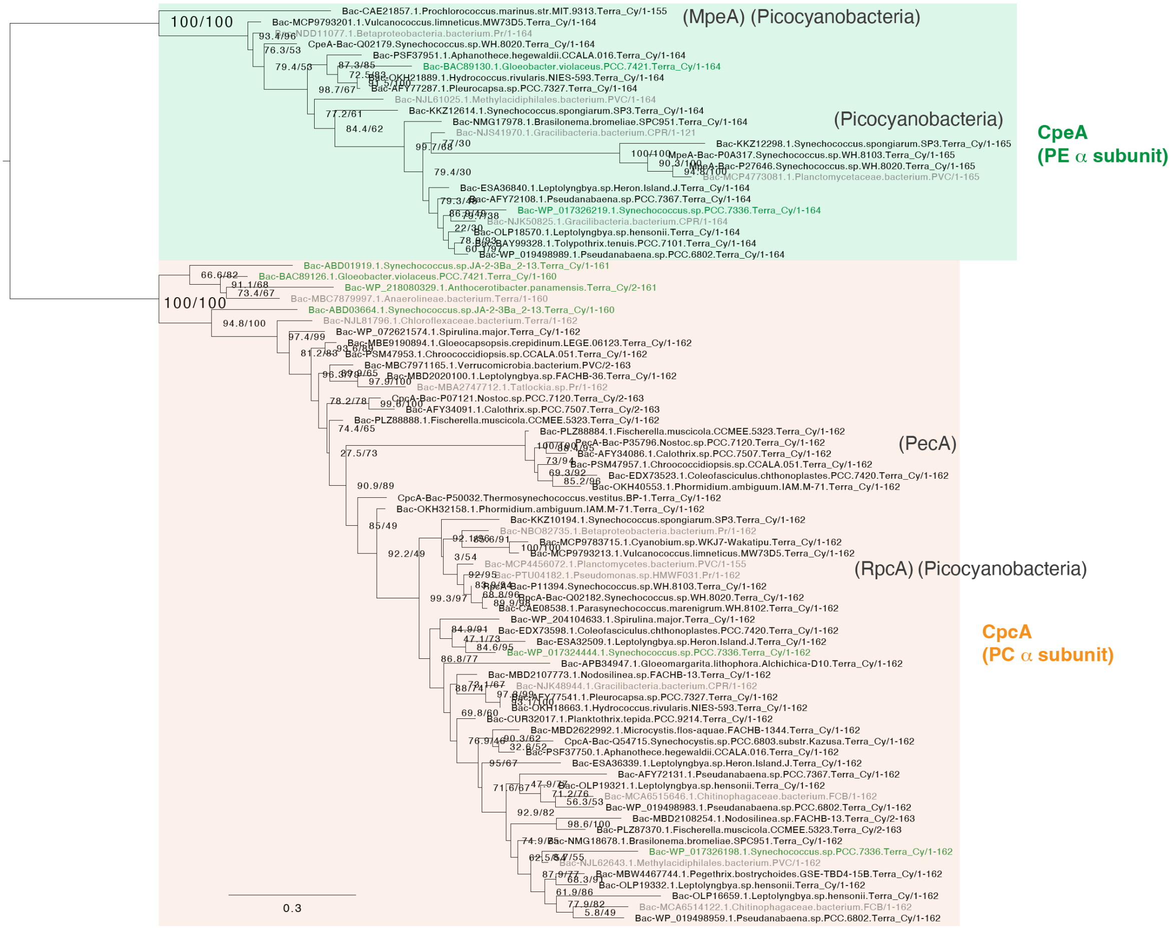

Supplementary Figure 3. Source data for Extended Data Figure 9: Maximum likelihood tree of CpcA and CpeA homologs (phycobiliprotein).

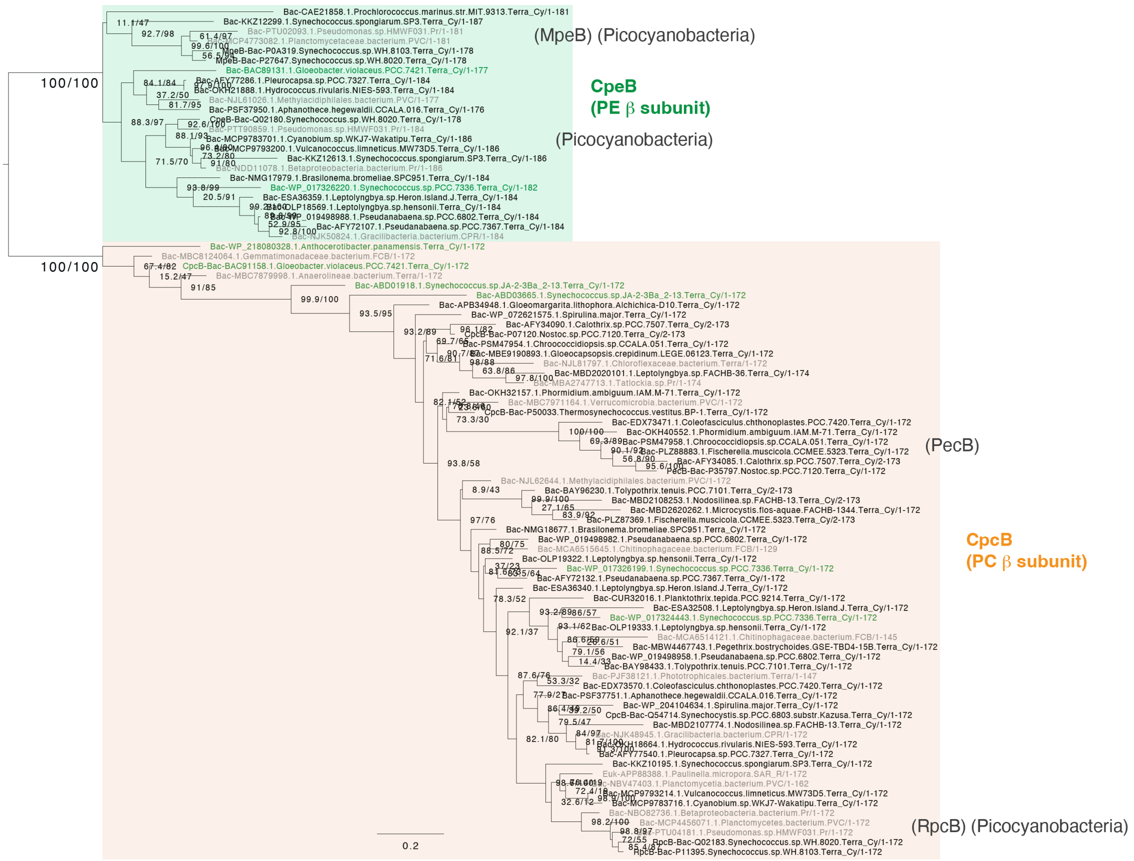

Supplementary Figure 4. Source data for Extended Data Figure 9: Maximum likelihood tree of CpcB and CpeB homologs (phycobiliprotein).

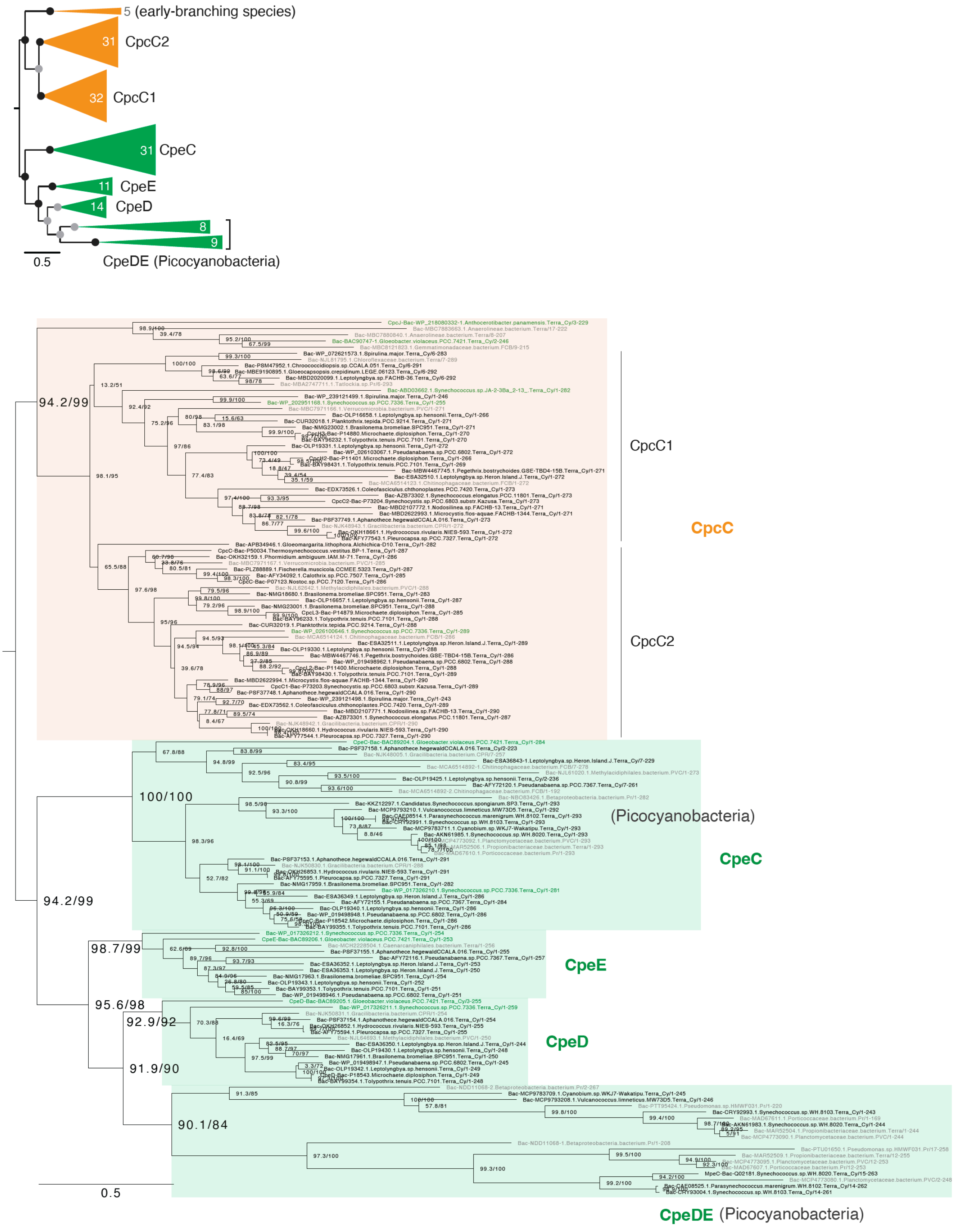

Supplementary Figure 5. Source data for Figure 3b and Extended Data Figure 9: Maximum likelihood tree of CpcC and CpeCDE homologs (rod linker).

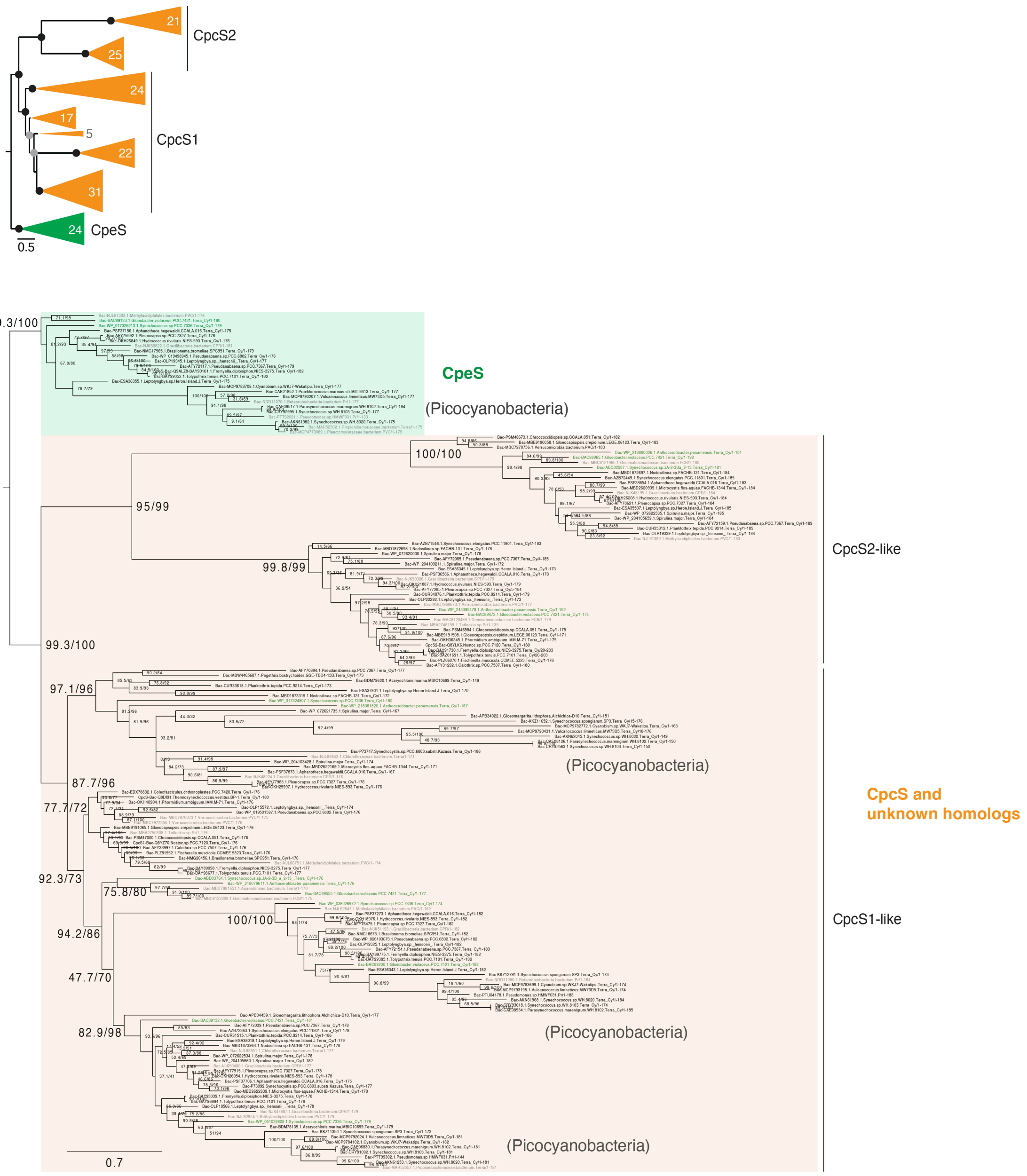

Supplementary Figure 6. Source data for Figure 3b and Extended Data Figure 9: Maximum likelihood tree of CpcS and CpeS homologs (lyase).

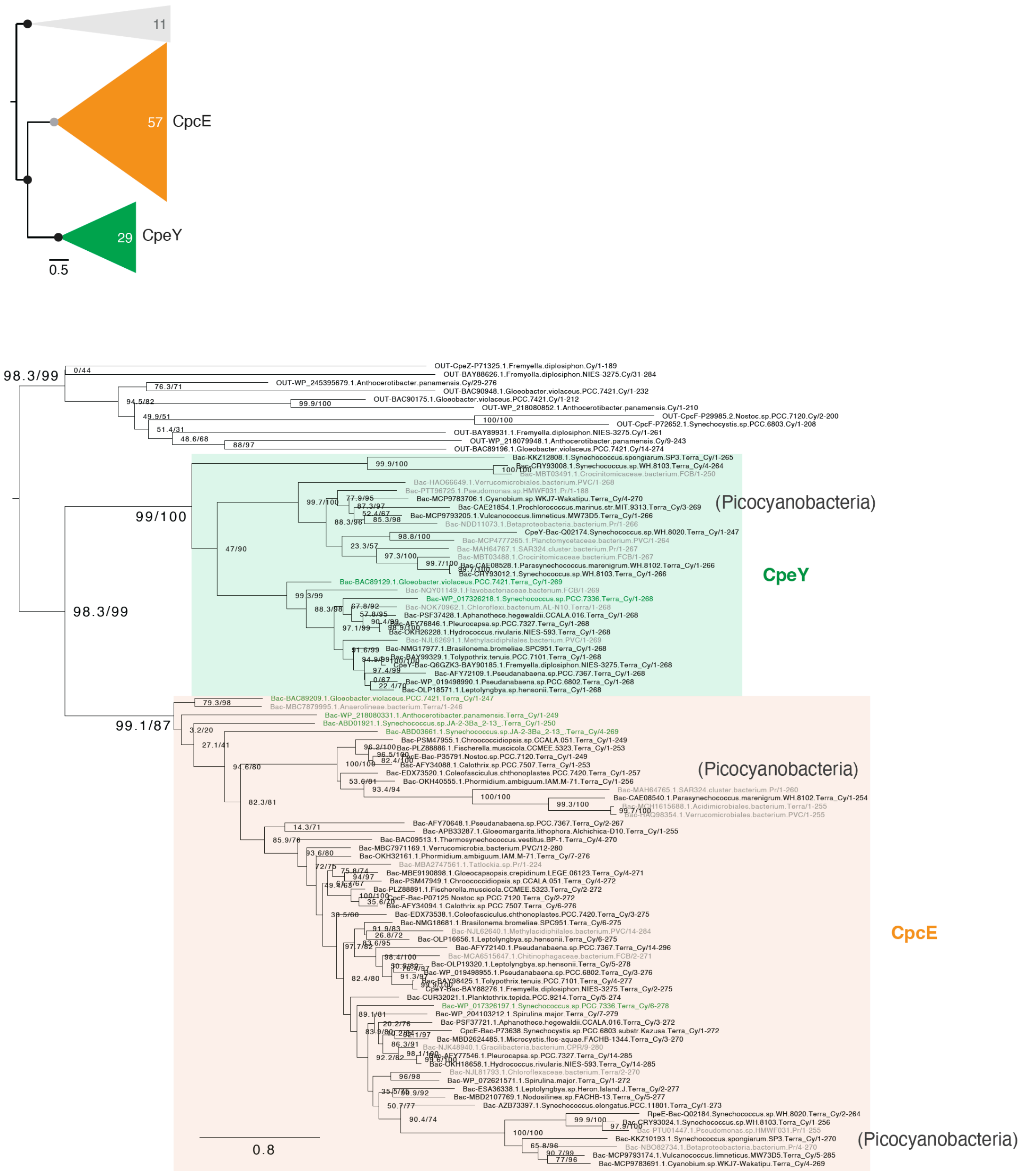

Supplementary Figure 7. Source data for Figure 3b and Extended Data Figure 9: Maximum likelihood tree of CpcE and CpeY homologs (lyase).

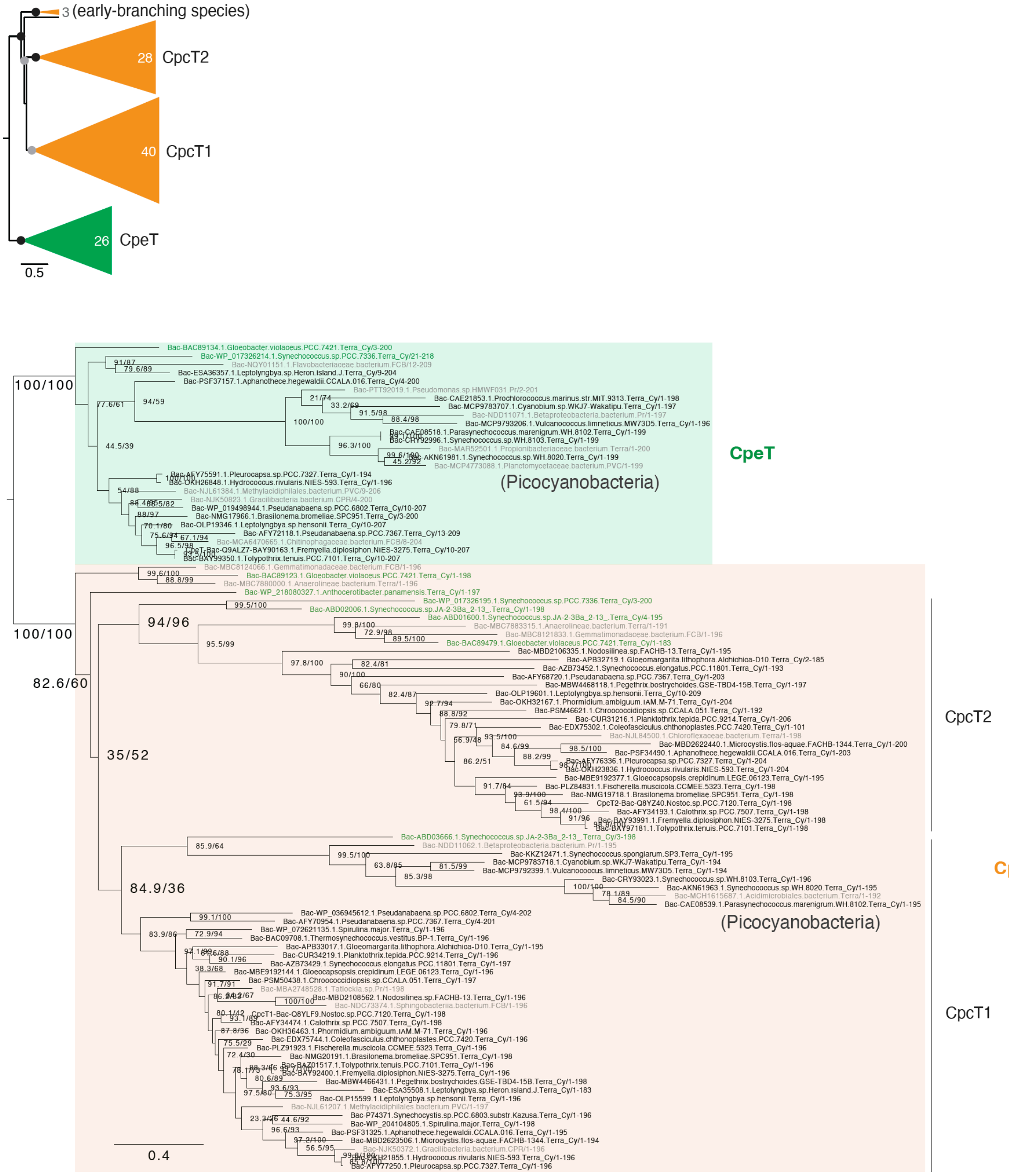

Supplementary Figure 8. Source data for Figure 3b and Extended Data Figure 9: Maximum likelihood tree of CpcT and CpeT homologs (lyase).
